# Policy, service, and training provision for women following a traumatic birth: an international knowledge mapping exercise

**DOI:** 10.1186/s12913-021-07238-x

**Published:** 2021-11-06

**Authors:** Gill Thomson, Magali Quillet Diop, Suzannah Stuijfzand, Antje Horsch, COST After birth Consortium, Joan G. Lalor, Wilson de Abreu, Valérie Avignon, Barbara Baranowska, Pelin Dikmen-Yildiz, Wissam El Hage, Yvonne Fontein-Kuipers, Antje Horsch, Susan Garthus-Niegel, Ernesto Gonzalez Mesa, Eleni Hadjigeorgiou, Maria Healy, Figen Inci, Gözde Gökçe İsbir, Ljiljana Jeličić, Sigfridur Inga Karlsdóttir, Georgia Kontosorou, Patricia Leahy-Warren, Julia Leinweber, Sylvia Murphy Tighe, Ursula Nagle, Jenny Patterson, Jessica Pehlke-Milde, Mirjana Sovilj, Claire Stramrood, Gill Thomson, Anastasia Topalidou, Maria Węgrzynowska

**Affiliations:** 1 School of Community Health & Midwifery, University of Central Lancashire, Preston, UK; 2 Institute of Higher Education and Research in Healthcare, University of Lausanne, Lausanne, Switzerland; 3 Department Woman-Mother-Child, Lausanne University Hospital, Lausanne, Switzerland; 4 School of Nursing and Midwifery, Trinity College Dublin, Dublin, Ireland

**Keywords:** Traumatic birth, Services, Policy, Training, Education, Survey

## Abstract

**Background:**

High numbers of women experience a traumatic birth, which can lead to childbirth-related post-traumatic stress disorder (CB-PTSD) onset, and negative and pervasive impacts for women, infants, and families. Policies, suitable service provision, and training are needed to identify and treat psychological morbidity following a traumatic birth experience, but currently there is little insight into whether and what is provided in different contexts. The aim of this knowledge mapping exercise was to map policy, service and training provision for women following a traumatic birth experience in different European countries.

**Methods:**

A survey was distributed as part of the COST Action “Perinatal mental health and birth-related trauma: Maximizing best practice and optimal outcomes”. Questions were designed to capture country level data; care provision (i.e., national policies or guidelines for the screening, treatment and/or prevention of a traumatic birth, service provision), and nationally mandated pre-registration and post-registration training for maternity professionals.

**Results:**

Eighteen countries participated. Only one country (the Netherlands) had national policies regarding the screening, treatment, and prevention of a traumatic birth experience/CB-PTSD. Service provision was provided formally in six countries (33%), and informally in the majority (78%). In almost all countries (89%), women could be referred to specialist perinatal or mental health services. Services tended to be provided by midwives, although some multidisciplinary practice was apparent. Seven (39%) of the countries offered ‘a few hours’ professional/pre-registration training, but none offered nationally mandated post-registration training.

**Conclusions:**

A traumatic birth experience is a key public health concern. Evidence highlights important gaps regarding formalized care provision and training for care providers.

**Supplementary Information:**

The online version contains supplementary material available at 10.1186/s12913-021-07238-x.

## Background

Perinatal mental health is a global public health issue due to its short and/or long term pervasive and negative impacts on women, infants, and families [1, 2]. A key cause of poor maternal health relates to a traumatic birth experience, defined as *‘the emergence of a baby from its mother in a way that involves events or care that cause deep distress or psychological disturbance, which may or may not involve physical injury, but results in psychological distress of an enduring nature’* [3](p.265). Another approach has been to conceptualise childbirth as “traumatic” if a (perceived) threat for the health of the mother and/or infant or severe physical injury occurred, based on the Diagnostic and Statistical Manuals (DSM) 5 Criterion A definition of a traumatic stressor [4]. The fact that a traumatic birth experience is subjectively defined [5] has meant prevalence data is inconsistent [6], with studies indicating between 9 and 50% of women experience their birth as traumatic [7–9].

Women who experience a traumatic birth report a range of psychological, social, cognitive, and behavioural related impacts. These include low self-esteem, relationships difficulties with their partner and infant,   social isolation, negative self-perceptions, early and unintended breastfeeding cessation, and difficulties with help seeking [10].. A traumatic birth is also linked to secondary tokophobia (a fear of childbirth following a previous traumatic birth), which can lead to women making difficult choices to prevent/not have any further children, or to have an intervention based birth in a future pregnancy [11]. A further implication of a traumatic birth relates to post-traumatic stress disorder (PTSD). PTSD is classified as a trauma- and stressor-related disorder which consists of four main symptom clusters, namely re-experiencing (e.g., flashbacks, nightmares), avoidance (of people, places and events that remind women of the birth), hyperarousal (such as being in a constant state of alert), and negative alterations in cognition and mood [4]. A recent systematic review and meta-analysis identified ~ 4% of women in general community samples, and ~ 19% of women in high-risk samples (i.e., previous history of mental illness, PTSD, premature birth, neonatal loss) go on to develop childbirth-related PTSD (CB-PTSD) [12].

While intrapersonal (i.e., history of mental health problem) and obstetric (i.e., clinical interventions) risk factors for CB-PTSD are reported [12], a further factor relates to a lack of, or poor relationships with care providers [13]. Women who have experienced a traumatic birth report feeling unsafe, abandoned, isolated, and unsupported [14, 15]. The need to train healthcare professionals into how birth can be experienced as traumatic has been highlighted [13, 15–17]. There have been recent moves in some countries, such as the UK, to introduce perinatal mental health training for post-qualifying maternity professionals [18]. However, to date, there is little known regarding mandated training within pre- or post-registration curriculum for maternity care professionals.

While specialist treatment options for PTSD, namely Eye Movement Desensitisation and Reprocessing (EMDR) and Cognitive Behavioural Therapy (CBT) are recommended, there are no recommended or standardised treatment options for women who have endured a traumatic birth [6, 19]. Arguably, offering an early intervention following a difficult and distressing birth would help to ameliorate women’s negative responses, and to prevent PTSD onset [20, 21]. In the UK [22] and Iceland [23], women are offered an after birth service: women who are distressed and traumatised by their birth can meet with maternity professionals to review their birth notes [24]. While women report variable experiences of these opportunities [24], women value being able to understand what happened and why, and to aid memory processing [23, 25]. A survey of UK after birth services [22] found wide heterogeneity in terms of whether the service was formally or informally provided, the times and timing of support, the different professionals involved (e.g., midwives vs. midwives and wider professionals) and the level of service provider training [22]. To date, the extent to which these service models reflect those in other international contexts is unknown.

The high number of women experiencing a traumatic birth, and the links between maternal CB-PTSD and poor developmental outcomes in infants [26] highlights a traumatic birth experience as a key public health concern. However, currently, there is a lack of insight into whether, what, or how support for a traumatic birth experience is provided in different contexts and settings [27]. In this study we report on a knowledge mapping exercise to help identify the policies, services,  and resources currently available for women following a traumatic birth [28]. We considered such insights could help identify ‘promising’ practices, or key service and policy gaps.

## Methods

### Aim

The aim of this knowledge mapping exercise was to map policy, service, and training provision for women following a traumatic birth within different European countries.

### Context

This knowledge mapping exercise was undertaken as part of the COST Action “Perinatal mental health and birth-related trauma: Maximizing best practice and optimal outcomes” (www.cost.eu/actions/CA18211). COST Action CA18211 is an EU-funded, multidisciplinary network of more than 160 researchers and clinicians from 33 countries with expertise in childbirth trauma and related topics, which was launched in October 2019. It closely collaborates with a network of relevant service user associations, as well as policy makers and health organisations across Europe and beyond. The objectives of this network are to produce, consolidate, and disseminate evidence to prevent, minimise, and resolve birth-related trauma, to optimise emotional and psychological outcomes for parents and families, as well as professionals working with this population, and to accelerate the translation of knowledge into best practices that can be shared internationally.

The authors put forward a call to all members of the COST Action to elicit interest in collaborating on the general topic of ‘after birth support following a traumatic birth’. Two meetings were held in January and February 2020 with ~ 20 representatives from different countries, during which it was agreed that the first step should be to map information across different European countries on the policies, types and extent of service provision for women following a traumatic birth experience, and professional training.

### Survey development and completion

In line with the knowledge mapping methodological guidance produced by Ebener et al. [28] the purpose of this exercise was to ‘bridge the gap’ in identifying the different health systems, i.e., policy making, service provision and resources in relation to a traumatic birth; *‘to understand how knowledge flows and where the assets and the gaps are’* (p.636). Ebener’s five-stage knowledge-mapping process was used [28], with the first stage ‘acquire the data’ involving a survey tool (see Additional file 1).  The survey was devised by the authors, with collaboration from those who attended the meetings.

The survey collected data in four main areas. First, ‘country level data’ comprised population level statistics including the number of inhabitants, number of births, and types of birth (setting, mode of birth) based on the most recent/verifiable data source; the number of maternity hospitals; and how the maternity system was funded. Second, ‘care provision’ included questions on whether there were any national policies or guidelines for the screening, treatment and/or prevention strategies for women following a traumatic birth experience, and if yes, to provide further details (authors, what the policies/guidelines are, and who they are provided for). This section also requested information on formal or informal services provided by maternity professionals (formal defined as service provision outside of normal/usual care that is regularly available and has allocated specific resources (personnel, time, etc.), and informal defined as service provision operating on an irregular basis, without allocated specific resources). If yes, respondents were asked to detail what the formal or informal service comprised, who provided the service, from which type of healthcare, whether it was a national or local initiative, and how the service was funded. A further question was included to capture whether women could be referred to specialist perinatal or mental health services. The last section captured whether there was any ‘training’ into traumatic birth for maternity professionals involved in perinatal care (i.e., midwives, obstetricians, obstetric nurses). This included questions on: 1) training provided as part of the national/general basic professional training/pre-registration curriculum and; 2) national mandatory requirements for post-registration training. If yes, respondents were asked to detail which professions, and how ‘much’ training was provided.

Similar to the examples of knowledge mapping detailed by Ebener et al. [28], this work involved engaging stakeholders and local experts. Individuals from the COST action (referred to as stakeholders in this paper) who were willing to participate were asked to collect data in consultation with local experts who had national knowledge of maternity care, perinatal mental health provision and/or pre- or post- registration training (and to detail who these individuals were) in their country. The stakeholders were asked to record any other comments (collected as part of their conversations with experts), which may be useful to help understand policies, practice, or training, in their country on the survey (see Additional file 1).

As this knowledge mapping exercise involved mapping existing policies, services, and training provision, rather than any individual level or evaluation-based data, full ethics approval was not required.

Data collection took place from March 2020 to February 2021. Stakeholders were issued with reminders (up to three) and asked to notify the authors if they were no longer able to collect the data.

### Data analysis

Data analysis followed the four analytical stages devised by Ebener and colleagues [28]. The first two stages are ‘manipulate data’ where the raw data are manipulated by basic analysis to produce ‘first-order’ data, and ‘store data’ where information is stored in secure files. This work involved all the survey data being transferred and stored into Excel files, using clear headings so any gaps or anomalies could be identified. During this stage follow-up emails were issued where needed, in attempts to collect a comprehensive data set. The next stage - ‘process data’ - involved the quantitative data being analysed using descriptive statistics (frequencies and percentages) for numerical (country level data) and dichotomous variables (yes/no). Any qualitative comments that helped to explain the stakeholders’ answers were also extracted and reported. In the final phase ‘visualize the data’, we produced visual maps to illustrate the knowledge available [28].

## Results

While participants from 23 countries originally agreed to participate, completed surveys were received from 18 countries; Belgium, Cyprus, England, France, Germany, Greece, Norway, Iceland, the Netherlands, Northern Ireland, Poland, Portugal, Ireland, Scotland, Serbia, Spain, Switzerland, and Turkey. The stakeholders and those consulted to complete the survey included midwives, psychologists, psychiatrists, obstetricians-gynaecologists, and nurses. In the following sections, the responses to the questions under the three key survey sections - ‘Country level data’; ‘Care provision’; and ‘Training for providers’ - are reported. As some stakeholders provided additional comments to help explain issues, such as the challenges in developing policies, or in delivering services following a traumatic birth experience, these have also been considered in the discussion.

### Country level data

Country level data from the 18 countries are presented in Table 1 (please contact lead author for references to data sources in each country). The data on the numbers of inhabitants and births per year was used to calculate the birth rate and showed variations from 7.8% in Greece to 14.2% in Turkey. The percentage of caesarean sections varied from 15.7% in the Netherlands to 56.8% in Greece. The percentage of home births varied from 0% in Cyprus, 2.1% in England, and was highest in the Netherlands at 12.7%. The ratio of maternity hospitals was also quantified to allow a comparison between countries. The highest ratio was 28 maternities per 1 million inhabitants in Cyprus and the lowest ratio was 2.4 in England. Most countries (72%) had a public and private maternity care system, compared with 28% of countries who had public care only.

**Table 1:** Country level data

	Number of inhabitants (in millions)^h^	Number of births per year	Birth rate (‰)	Average % of caesarean sections per year across country	% of home births per year	Care system^j^	Number of maternity hospitals^k^	Ratio of maternity hospitals (number per 1 million inhabitants)
Belgium	11.49	115,565	10.1%	21%	0.53%	1	104	9
Cyprus	1.2	9548^a^	10.7%^b^	54%	0%	2	34^c^	28
England	56	625,651	11.2%	29%	2.1%	1	134	2.4
France	67	753,000	11.2%	19.7%	0.6%	2	513	7.7
Germany	83.02	784,901	9.5%	30.5%	1.3%	2	672	8.1
Greece	10.8	83,763	7.8%^a^	56.8%	< 1%	2	107^d^	10.3
Iceland	0.35	4448	12.6‰	16.1%	1.8%	1	7	20
Ireland	4.76	61,084	12.8‰	33.8%	0.2%	2	19	4
Netherlands	17.43	161,720	9.3‰	15.7%	12.7%	1	75	4.3
Northern Ireland	1.91	20,814	10.9‰	32%	0.22%	2	17^e^	8.9
Norway	5.38	54,407	10.1‰	15.9%	0.41%	2	47^f^	8.7
Poland	38.41	389,603	10.1‰	44.7%	0.2%	2	387	10.1
Portugal	10.28	86,256	8.4‰	32.5%	1%	2	238	23.2
Republic of Serbia	7	63,975	9.2‰	32.2%	0.15%	2	58	8.3
Scotland	5.5	48,912	8.9‰	34.5%	1.17%	1	43^g^	7.8
Spain	47.33	359,770	7.6‰	26.7%	0.32%	2	511	10.8
Switzerland	8.6	86,172	10.0‰	32.0%	1.03%	2	87	10.1
Turkey	83.15	1,183,652	14.2‰	53.1%	0.9%	2	1329	16

^a^In the government-controlled area (South)

^b^Data was collected direct from the stakeholders

^c^5 public hospitals and 29 maternity private clinics

^d^64 public maternity units and 43 private maternity units

^e^8 maternity hospitals and 9 Midwife-led units (6 Alongside MLUs & 3 Free Standing MLUs – Reconfiguring due to COVID-19 currently there are - 6 AMUs and 1 FMU with other units planned to reopen)

^f^42 maternity clinics and 5 maternity wards

^g^18 obstetric units, 19 freestanding midwife-led units and 6 alongside midwife-led units

^h^Country level data was based on the most recent available census, at the time of data collection. Some data were rounded to two decimal places. ^i^The data used to calculate *birth rate* and *ratio of maternity hospitals* were sometimes collected for different reference years. ^j^Care system: 1 = public care only; 2 = public and private care. ^k^ There were inconsistencies in how data was reported – some provided the numbers of maternity hospitals, whereas others detailed the different levels of provision, i.e. numbers of maternity units, consultant led units, etc.

### Care provision

#### National policies or guidelines

Apart from the Netherlands, there was no other country who had a national policy or guidelines for screening, treating, or preventing psychological issues linked to a traumatic birth experience. While the stakeholder from Scotland indicated there were policies to prevent women from having a traumatic birth experience, they related only to physical trauma (i.e., to reduce anal sphincter injury or stillbirth rate). Other stakeholders, such as those from Poland, reported on more general policies to improve birth outcomes and maternal wellbeing, such as “[ …] *pre-birth education aimed at reducing anxiety associated with labour and early motherhood, but nothing* […] *that would specifically address the prevention of traumatic birth*” (Poland stakeholder). Likewise, there were general guidelines in France for the screening of postpartum psychological disorders and also “[ …] *to avoid obstetric complications of childbirth* [ …]” , but no national policies or guidelines specifically related to the screening, treatment and/or prevention of a traumatic childbirth experience.

The Dutch guideline was noted to have been  recently published (2019) [29], and the stakeholder highlighted two important recommendations for screening:

*- "Ask women how they have experienced labor and delivery: in the first week after birth, at the 6 weeks check up appointment, and at the beginning of a new pregnancy. – Make use of a validated screening instrument for postpartum PTSD in women who report a traumatic delivery experience and in women who are at increased risk of developing postpartum PTSD”.*

The guideline also detailed treatment options for women with traumatic experiences, who had CB-PTSD symptoms or a CB-PTSD diagnosis, as commented by the Dutch stakeholder: “*In case of PTSD: treat as you would treat PTSD after other trauma, namely: psychoeducation combined with EMDR or trauma-focused CBT”.* It also considered prevention in terms of how to care for women during childbirth *“Aim for continuous 1-on-1 care, for example by a trained lay person not involved in medical care and decision making (e.g. Doula)”* as well as an early intervention such as expressive writing to help women process their memories about the birth “*Consider a short expressive writing exercise aimed at emotions, thoughts and initial expectations about labor and delivery”*.

#### Service provision

All countries, except for Cyprus and Turkey, had some form of service provision. Thirty-three percent of the countries (England, Iceland, Northern Ireland, Ireland, Scotland, and Switzerland) indicated that formal services were available, 78% had informal services, and 89% were able to offer referrals to specialist services. The six countries with formal services also had informal services and referral options for specialist provision.

It is important to note that formal service provision was not always routinely provided for all women. For example, in Scotland, it was reported “*In most NHS* [national health service] *boards a follow-up debrief is offered with a consultant obstetrician for women whose births were considered objectively traumatic, i.e. emergency CS, large blood loss, 3*^*rd*^ *degree tear” *(Scotland stakeholder), suggesting it was only available for those with pre-supposed clinical needs. Formal services were also not available in all the country’s maternity hospitals. For example, in Ireland, the stakeholder reported that only two of the maternity units provided ‘*a birth reflection type of service, where women can discuss their birth experience. One service is a dedicated collaborative clinic*”. Whereas the Icelandic stakeholder stated there were *“only two counselling clinics for women experiencing traumatic birth”.* Similarly, in Northern Ireland it was reported “*services vary across the HSCTs* [health and social care trusts] *in Northern Ireland and include - support, debriefing & limited perinatal mental health service provision for some women in individual Trusts where they have access to therapy”* (Northern Ireland stakeholder).

One hospital in Switzerland was currently in receipt of short-term funding to provide a formal service to women and their partners. Moreover, the formal provision offered in England was reported to be variable and insufficient: *“Some Trusts provide a formal after birth debriefing service for women who have had a difficult/distressing/complicated birth (but lack of governance/procedures to underpin service delivery)”* (England stakeholder)*.*

Formal services were provided mainly by midwives, either solely or in conjunction with obstetricians in Switzerland and Scotland (33%), or with obstetricians and/or mental health counsellors in England, Iceland, Northern Ireland and Ireland (67%). For example in Ireland: “*The perinatal birth trauma service is a collaborative service facilitated by an advanced midwife practitioner, a psychiatrist and a psychologist who liaise closely with a named obstetrician”* (Ireland stakeholder)*.* Nearly all available formal services were reported to be local initiatives (83%), except in Scotland. The majority of services were provided in hospitals, and were publically funded (83%), although in some countries, this was mixed. For example, in Ireland, three services were publically available within public and privately funded maternity services.

#### Training for providers

Seven countries (39%), i.e., Cyprus, France, Iceland, the Netherlands, Northern Ireland, Portugal, and Scotland indicated that training into traumatic birth/CB-PTSD was part of the national/general basic professional training/pre-registration curriculum for some of the key professionals involved in perinatal care. This training was provided for midwives in all countries, but also for obstetricians in France, medical doctors in Iceland, and obstetric nurses in the Netherlands. However, there was very little basic education, i.e., only a few hours’ training provided for some curricula in all these countries. In Iceland, the stakeholder reported that training into traumatic birth was not included in specific courses, *“[ …] but it is discussed in some modules”*. Moreover, some stakeholders reported optional courses and/or local ad hoc training provided for midwives, psychologists and obstetricians/doctors in the Netherlands and Norway, for maternity healthcare professionals in Ireland, and for psychiatrists and psychologists in Greece. Regarding post-registration training into traumatic birth, there was no national mandatory requirement for maternity care professionals in any country.

**Figure 1:**
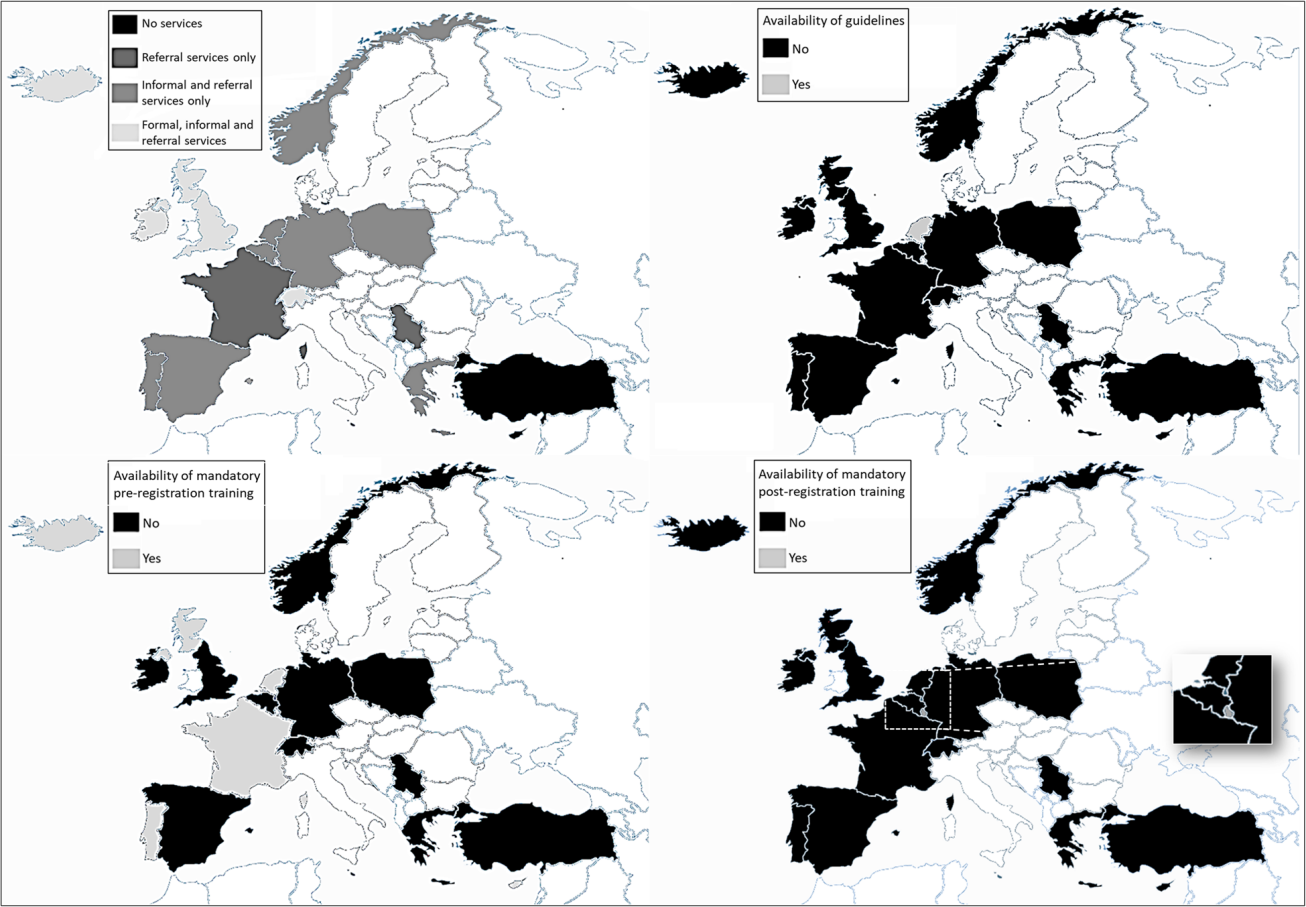
Fig. 1 Policies, services, and training provision for traumatic birth

In line with Ebener’s final ‘visualize data’ stage [28], the resulting knowledge map of the presence/absence of national policies or guidelines, formal service provision, and training for providers are presented in visual maps (see Fig. 1).

## Discussion

The aim of this knowledge mapping exercise was to map policy, service, and training provision for women following a traumatic birth experience within different European countries. The findings from 18 countries across Europe revealed that only one country had national policies or guidelines in place regarding the screening, treatment, or prevention of a traumatic birth experience. Formal services offered to women experiencing a traumatic birth were only available in six countries (33%). However, the stakeholders indicated that this type of service was informally provided in most included countries (78%), with a possibility for women to be referred to specialist perinatal or mental health services (89%). The formal services were generally publically funded, provided in hospitals, and by midwives. More than a third of the countries (39%) offered training into traumatic birth as part of national basic professional training for maternity professionals. None of the countries had any national mandatory requirement to receive post-registration training into traumatic birth/CB-PTSD.

The Dutch multidisciplinary guideline recommends the use of validated screening tools, such as the Primary Care PTSD screen for the DSM-5 [30] to identify women who are experiencing CB-PTSD symptoms. However, although this questionnaire assesses PTSD symptoms following a stressful/traumatic event, it does not specifically assess CB-PTSD symptoms. The City Birth Trauma Scale (City BiTS) [31], which has already been validated in several languages, might be more appropriate for use in routine clinical practice. A systematic screening procedure is essential for the detection of women reporting a traumatic childbirth experience, in order to promote their access to appropriate care. This is particularly important as women often avoid professional contact following a traumatic childbirth [10], may lack insight into how to access help [9, 32], and may be reticent to disclose poor mental health for fear of repercussions and stigma [33]. Women may also not realise they are experiencing the effects of CB-PTSD due to being overwhelmed with new motherhood [20], and/or due to symptoms manifesting at a later point [34], and after women have been discharged from maternity services. A further complication also relates to CB-PTSD symptoms being misdiagnosed as post-natal depression [35]. These issues highlight a need for women to receive further information, i.e. within discharge packs, to help raise awareness of CB-PTSD symptomatology and to encourage help-seeking, such as via primary care.

The lack of formalised provision for women following a traumatic birth raises obvious concerns over availability and sustainability, as indicated by a comment within the Norway survey “*There are some good offers here and there, but this is mostly based on passionate souls*.“  Our finding of formal provision not being routinely provided for all women is also in line with a UK-based study showing that women were more likely to self-refer (79.6%), rather than be referred via routine screening (11.1%), or according to obstetric criteria (27.8%) [22]. Several stakeholders also indicated the availability of formal services for women following childbirth, but most of the time, they were not specifically dedicated to birth trauma. Instead, an allocated specific budget was commonly devoted to women with objective obstetrical complications (i.e., emergency caesarean section, stillbirth, etc.), with depressive symptoms, and/or experiencing family, social or personal complexities, rather than women’s subjective experience of their childbirth having been traumatic. A further challenge was the evident controversy about whether childbirth can be considered a traumatic event and to lead to CB-PTSD onset. The French stakeholder related *“[…] some feedback mentioning that the entity of birth-related PTSD is seen by some trainers as controversial, considering that PTSD is most probably related to another event than traumatic birth. As if birth cannot be traumatic!*”. This lack of clarity could be due to different terms, such as traumatic birth [5, 10, 14, 36] or negative birth experience [37–39] being used interchangeably, as well as trauma being used in the obstetric/medical literature to indicate physical rather than psychological trauma. The denial of childbirth as a potentially traumatic event is obviously a concern, as without this recognition, dedicated policies, appropriate service provision, and training are unlikely to follow. Further work to raise awareness of the prevalence, indicators, and impacts of this phenomenon is therefore crucial.

Service provision was often described as an interview, during which women could discuss their childbirth experience, but others referred to it as debriefing, counselling, information and/or reflective listening sessions. This is reflective of wider arguments concerning the lack of definition as to what after birth services comprise [40]. In the UK, the National Institute of Health and Clinical Evidence postnatal guidelines stipulate that women should not be offered a debrief, rather to have a conversation with their midwife about their labour and birth [41]. This is due to Cochrane reviews concluding there is insufficient evidence for debriefing interventions (e.g. [42]), although important to note that this conclusion is based on heterogenous intervention designs which target different populations (i.e., women with perceived clinical and/or psychological need) [24]. In the UK, Birth Trauma Resolution therapy is accredited by the Royal College of Midwives for use within clinical practice [43], but as yet, there is no formal evidence of its effectiveness within a perinatal population. Further work to develop effective and evidence-based after birth support is needed [9]. In the Netherlands, insurance does not cover the service provision of many midwives for women following a traumatic birth experience because “*their status [is] not official [ …*], *[and] [ …] controversial (i.e., professional organisations of psychologists do not approve of them offering, for example, EMDR, while the midwives’ association has accredited the training to become such a counsellor), and the background of these providers is very diverse*”.  As midwives are at the forefront of providing care for pregnant and postpartum women, and women often want to receive care from maternity professionals following a traumatic birth experience [9], the implementation of a validated, specialized and nationally recognized training for midwives, as well as other maternity healthcare professionals is essential. At the same time, discussion of professional responsibilities and boundaries, e.g., a detailed discussion about the birth experience and screening for trauma-related psychological symptoms by maternity professionals as part of the after birth service but referral of those with trauma-related psychological symptoms to specialist perinatal mental health services, should take place on a national level with relevant professional organisations.

The strengths of this work are it is the first mapping exercise to explore whether there are any national guidelines, services, or training provision for women who have experienced a traumatic birth in different European contexts. Such evidence helps to identify promising practices, key gaps, and to inform future research priorities. The limitations relate to a lower response rate than intended. Not all European countries are represented in this data set, and while originally 23 countries agreed to participate, and despite calls for other country representatives, overall, only 18 were included. All the included countries have high income-status, and the evident gaps in these contexts would suggest the situation could be even worse in middle or low-income countries. The survey only collected information on what was available, rather than any individual level or evaluation-based data. As many of the countries provided ‘some’ form of service provision (albeit informally), research to elicit further insights into what and how services are provided, as well as the outcomes and benefits for women is needed. This work could help identify key mechanisms of effectiveness and to progress towards developing standardised, evidence-based interventions to improve outcomes for women and families.

## Conclusion

This mapping exercise into policy, services, and training associated with a traumatic birth experience within 18 different European countries highlighted a lack of national policy guidance on the prevention, care, and treatment of a traumatic birth experience, an absence of formal after birth services, as well as a lack of mandatory pre- and post-registration training. Potential barriers to formalized and mandated provision pertain to uncertainties regarding the definition of traumatic birth, a lack of evidence-based early interventions for women following traumatic birth, and a lack of public funding of after birth care services. Further work is needed to determine the essential ingredients of effective, evidence-based after birth care provision, the development of policy guidance, as well as professional training, to optimize maternal and familial wellbeing.

## Supplementary Information

**Additional file 1.** Survey. Mapping of service provision for women following a traumatic birth. Survey tool to collect the information.

## Data Availability

The references and web links for country level data, and the full dataset used and/or analysed during the current study are available from the corresponding author on reasonable request.

## Abbreviations

CB-PTSD: Childbirth related post-traumatic stress disorder
CBT: Cognitive Behavioural Therapy
CS: Caesarean section
DSM: Diagnostic and Statistical Manuals
EMDR: Eye Movement Desensitisation and Reprocessing
NHS: National Health Service
PTSD: Post-traumatic stress disorder
UK: United Kingdom
