# Supplementary material for: Policy, service, and training provision for women following a traumatic birth: an international knowledge mapping exercise
Source: BMC Health Serv Res. 2021 Nov 6;21:1206. doi: 10.1186/s12913-021-07238-x (PMC8571982; doi:10.1186/s12913-021-07238-x)
Supplement: Supplementary file 1 — Additional file 1. Survey. Mapping of service provision for women following a traumatic birth. Survey tool to collect the information. [file 12913_2021_7238_MOESM1_ESM.docx]

Additional File 1: **Survey - Mapping of service provision for women following a traumatic birth**

The aim of this short survey is to provide some general information into the policies, training and types and extent of formal service provision for women following a traumatic birth^[[1]](#footnote-1)^within different EU countries. Please note that we only require brief summaries in response to these questions to enable us to compare information from the different cultural settings. This survey should be completed by designated country representative(s) after consultation with experts e.g. national organisations, key experts. While these ‘experts’ will vary by country, we expect these to have national knowledge of maternity care, perinatal mental health provision and/or pre-registration training.

Once you have collected data from all relevant experts, please provide a summary of their responses under each question below. The deadline for completion is **Friday 29^th^ May 2020.**

1. **Survey completion:** Please detail the number and professional roles of those consulted to complete this survey

2. **Country level data:**

a) Please state the name of your country:

b) Please provide details on:

*(Please state the year of data collection and where you obtained the information (including reference/web link as appropriate) for each of the following information requests. If any of the information requested is not available for your country, please state: “Not reported”)*

1. Number of inhabitants:
2. Number of births per year:
3. What is the number/ % of cesarean sections per year :
4. What is the number/% of home births per year:
5. Our maternity care system is (please check the box as appropriate):

| Public care only |  |
| --- | --- |
| Public and private care |  |
| Private care only |  |

1. How many maternity hospitals are there in your country:
2. Please report any further details on the maternity care system in your country you believe are important below:

3. **Care provision**

a) In your country, are there any national policies or guidelines for the *screening* of women following a traumatic birth? Yes / No

1. If yes – please provide details of the authors, what the policies/guidelines are, and who they are provided for.
2. In your country, are there any national policies or guidelines for the *treatment* of women following a traumatic birth? Yes / No
3. If yes – please provide details of the authors, what the policies/guidelines are and who they are provided for.

c) In your country, are there any national policies or guidelines to *prevent* women from having a traumatic birth? Yes / No

1. If yes – please provide details on the authors, what the policies/guidelines are and who they are provided for.

d) In your country, are any formal services^[[2]](#footnote-2)^offered for women following a traumatic birth? Yes / No

If yes:

1. What types of formal services^2^ are offered?
2. Who provides the services (e.g. midwives, obstetricians, psychologists) and from which types of healthcare provision (e.g. public or private sector, hospital, community health board, etc)
3. Are these formal services a national or a local initiative?
4. How are these services funded – public funding, private funding, health care insurance

e) In your country, are there any informal services (ad hoc discussions or debriefing with midwives and/or obstetricians) offered for women following a traumatic birth? Yes / No

f) In your country, is it possible to refer women following a traumatic birth to specialist perinatal or mental health services? Yes / No

4. **Training for providers**

a) In your country, is training into traumatic birth/birth related PTSD part of the national/general basic professional training/pre-registration curriculum for any of the key professionals involved in perinatal care (for midwives, obstetricians, obstetric nurses)? Yes / No

If yes:

1. which profession (s)
2. How ‘much’ training is provided, e.g. an hour, one module (if known)
3. In your country, are there any national mandatory requirements (e.g. via the professional bodies) for maternity care professionals (midwives, obstetricians, obstetric nurses, etc) to receive post-registration training into traumatic birth/birth related PTSD Yes / No

If yes:

1. which profession (s)
2. How ‘much’ training is provided, e.g. an hour, one module (if known)
3. How often is this training provided?

If you have any other comments which may be useful to help understand policies, training, or practice in your country – please detail here:

1. **Traumatic birth** refers to a woman’s subjective experience of her birth. [↑](#footnote-ref-1)
2. **Formal** **service provision** relates to service provision that is outside of the normal/usual (ad hoc) care a pregnant or postnatal woman would receive. It is regularly and permanently available and a specific budget (personnel, time, etc.) is allocated to it. [↑](#footnote-ref-2)
